# Supplementary material for: CrebA regulation of secretory capacity: Genome-wide transcription profiling coupled with in vivo DNA binding studies
Source: bioRxiv. 2025 Jun 17:2025.06.12.659381. Preprint. [Version 1] doi: 10.1101/2025.06.12.659381 (PMC12262295; doi:10.1101/2025.06.12.659381)
Supplement: Supplement 1 [file NIHPP2025.06.12.659381v1-supplement-1.pdf]

bioRxiv preprint doi: https://doi.org/10.1101/2025.06.12.659381; this version posted June 17, 2025. The copyright holder for this preprint (which was not certified by peer review) is the author/funder, who has granted bioRxiv a license to display the preprint in perpetuity. It is made available under aCC-BY 4.0 International license.

Supplemental Figure Legends

Figure S1. Analysis of *CrebA* and SPCG expression across tissues from late-stage (stage 13-16) WT single cell RNA sequencing (scRNA-seq). A. UMAP showing that all the major cell types are represented

bioRxiv preprint doi: https://doi.org/10.1101/2025.06.12.659381; this version posted June 17, 2025. The copyright holder for this preprint (which was not certified by peer review) is the author/funder, who has granted bioRxiv a license to display the preprint in perpetuity. It is made available under aCC-BY 4.0 International license.

in the data (Peng et al., 2024). **B.** Violin plot of CrebA expression across tissues: tissues expressing highest levels are on the left, lowest on the right. **C.** In situ hybridization of *CrebA* (left panels) and CrebA immunostaining (right panels) at the stages captured by the late scRNA-seq analysis. The first and third rows are lateral views, the second and fourth are dorsal-ventral views. Abbreviations: SG – salivary gland, Tr – trachea, Es – esophagus, PV – proventriculus, Ph – pharynx, HG – hindgut, GL – glia, FB – fat body, St – stage. **D.** Late embryonic expression levels of *CrebA*, most known SPCGs, and three other known CrebA targets across late stage embryonic tissues.

Figure S2. Analysis of CrebA mutant scRNA-seq replicates and of the cell types captured in distinct clusters. A. UMAP showing the overlay of cells in *CrebA* mutant samples from biological replicas 1 and 2 after harmonization. **B.** UMAP from the merged *CrebA* mutant data showing the 40 identified clusters. **C.** UMAPs of *CrebA* mutant scRNA-seq data showing which cells express top apodeme markers (*CG7296*, *TwdlL*, *TwdlB*). **D.** UMAPs of WT scRNA-seq data showing which cells express the top optic lobe markers (*E(spl)m5-HLH*, *SoxN*, *Obp99a*).

Figure S3. Gene expression profiles across different tissues do not change much in *CrebA* null versus WT early embryos. Graph showing the Spearman correlation in gene expression profiles across all tissues in WT and *CrebA* mutant scRNA-seq data plotted against relative levels of *CrebA* RNA expression in different tissues.

Figure S4. Analysis of gene expression changes in *CrebA* null versus WT in late embryos and in tissues that do not express *CrebA*. A. UMAP from late stage *CrebA* null embryonic scRNA-seq showing that all major cell types found in the data. **B.** Gene set enrichment analysis (GSEA) reveals gene set categories enriched in the WT relative to *CrebA* null glia and hindgut at late stages (stage 13-16). **C.** Gene set enrichment analysis (GSEA) reveal gene set categories enriched in the glia and hindgut in *CrebA* null tissues relative to WT at late stages. **D.** GSEA analysis of WT versus *CrebA* null early gene expression in tissues that express the lowest levels of *CrebA* at early stages (stage 10-12). **E.** GSEA analysis of *CrebA* null versus WT early gene expression in tissues that express the lowest levels of *CrebA* at early stages.

bioRxiv preprint doi: https://doi.org/10.1101/2025.06.12.659381; this version posted June 17, 2025. The copyright holder for this preprint (which was not certified by peer review) is the author/funder, who has granted bioRxiv a license to display the preprint in perpetuity. It is made available under aCC-BY 4.0 International license.

Figure S5 (two pages). βGal and SPCG expression in embryos using the different Gal4 lines to drive either *lacZ* or *CrebA* expression in the embryo. A. Expression of three SPCGs in Oregon R embryos. **B.** Expression of the same three SPCGs in embryos using the *en*-Gal4 driver to drive UAS-*CrebA* expression in the embryo. **C.** Expression of βgal using the *mef2*-Gal4 driver to drive UAS-*lacZ* expression in the embryos. **D.** Expression of βgal using the *twi*-Gal4 driver to drive UAS-*lacZ* expression in the embryo. **E.** Expression of three SPCGs in embryos using the *mef2*-Gal4 driver to drive UAS-*CrebA* expression in the embryos. **F.** Expression of three SPCGs in embryos using the *twi*-Gal4 driver to drive UAS-*CrebA* expression in the embryos. **G.** Expression of βgal (top two rows) using the *elav*-Gal4 driver to drive UAS-*lacZ* expression in the embryos. Note that βgal is expressed in both the CNS and PNS, although we did not detect CrebA expression in the PNS using this same driver (see Figure 4E). **H.** Expression of βgal using the *nos*-Gal4 driver to drive UAS-*lacZ* expression in the embryo. Note that although βgal expression shows high level background staining with this driver, we did not detect high CrebA background staining with this driver (see Figure 4F). **I.** Expression of three SPCGs in embryos using the *elav*-Gal4 driver to drive UAS-*CrebA* expression in the embryos. **F.** Expression of two SPCGs in embryos using the *nos*-Gal4 driver to drive UAS-*CrebA* expression in the embryos. Top rows of embryos for each antibody or probe are lateral views. Bottom rows of embryos for each antibody or probe are mostly ventral views. The left most column in all sets are st 11 – 12, the middle column in all sets are stage 13. The right most column in all sets are ~ st 15. Arrows highlight ectopic expression of lacZ/βGal and mRNAs. Abbrev. M = mesoderm, CVM = circular visceral mesoderm, S = somatic muscle, ME = midgut endoderm, CNS = central nervous system, GC = germ cells. For each set **A - F**, left panels are ~stage 11, middle panels are ~stage 13 and right panels are ~stage 15.

Figure S6. Overlap of genes that go down in *CrebA* nulls found using microarray and genes that go down in *CrebA* null SGs (both early and late stages) with scRNA-seq and with SPCGs.

Figure S7. Number of CrebA binding peaks observed with 0, 1 and 2 nearest genes whose 5' ends map within 1 kb. Note that a significant number of CrebA peaks have no nearby genes and only a small fraction have two nearby candidate genes.

bioRxiv preprint doi: https://doi.org/10.1101/2025.06.12.659381; this version posted June 17, 2025. The copyright holder for this preprint (which was not certified by peer review) is the author/funder, who has granted bioRxiv a license to display the preprint in perpetuity. It is made available under aCC-BY 4.0 International license.

Figure S8. Enriched motifs in genes bound and whose expression goes down with loss of *CrebA* and the SG-expressed TFs that are known to bind each motif. A. The top enriched motif found in CrebA bound genes that go down in *CrebA* null embryos matches the consensus motif for TFs expressed in the SG (green bars). Three of these (CrebA and Max) have enriched SG expression (dark green). **B.** The second most enriched motif found in genes whose expression is reduced in *CrebA* mutants matches the consensus motif for TFs expressed in the SG (green bars). Sage has enriched SG expression (dark green).

Supplemental Figure Legends

Table S1. **Marker genes for the clusters in stage 10-12 *CrebA* mutant scRNA-seq atlas.**

Table S2. Differentially expressed genes between stage 10-12 wild-type and *CrebA* mutant cell types.

Table S3. GSEA results for stage 10-12 wild-type cell types.

Table S4. GSEA results for stage 10-12 *CrebA* mutant cell types.

Table S5. Downstream targets of CrebA
